# Protein profiling of the dimorphic, pathogenic fungus, *Penicillium marneffei*

**DOI:** 10.1186/1477-5956-6-17

**Published:** 2008-06-04

**Authors:** Julie M Chandler, Erin R Treece, Heather R Trenary, Jessica L Brenneman, Tressa J Flickner, Jonathan L Frommelt, Zaw M Oo, Megan M Patterson, William T Rundle, Olga V Valle, Thomas D Kim, Gary R Walker, Chester R Cooper

**Affiliations:** 1 Proteomics Research Group, Department of Biological Sciences, Youngstown State University, Youngstown, OH 44555-3601, USA; 2 Department of Chemistry, Youngstown State University, Youngstown, OH 44555-3663, USA; 3 Department of Chemistry, Rochester Institute of Technology, One Lomb Memorial Drive, Rochester, NY 14623-5603, USA; 4 Department of Chemistry, University of Cincinnati, Cincinnati, OH 45221-0172, USA

## Abstract

**Background:**

*Penicillium marneffei *is a pathogenic fungus that afflicts immunocompromised individuals having lived or traveled in Southeast Asia. This species is unique in that it is the only dimorphic member of the genus. Dimorphism results from a process, termed phase transition, which is regulated by temperature of incubation. At room temperature, the fungus grows filamentously (mould phase), but at body temperature (37°C), a uninucleate yeast form develops that reproduces by fission. Formation of the yeast phase appears to be a requisite for pathogenicity. To date, no genes have been identified in *P. marneffei *that strictly induce mould-to-yeast phase conversion. In an effort to help identify potential gene products associated with morphogenesis, protein profiles were generated from the yeast and mould phases of *P. marneffei*.

**Results:**

Whole cell proteins from the early stages of mould and yeast development in *P. marneffei *were resolved by two-dimensional gel electrophoresis. Selected proteins were recovered and sequenced by capillary-liquid chromatography-nanospray tandem mass spectrometry. Putative identifications were derived by searching available databases for homologous fungal sequences. Proteins found common to both mould and yeast phases included the signal transduction proteins cyclophilin and a RACK1-like ortholog, as well as those related to general metabolism, energy production, and protection from oxygen radicals. Many of the mould-specific proteins identified possessed similar functions. By comparison, proteins exhibiting increased expression during development of the parasitic yeast phase comprised those involved in heat-shock responses, general metabolism, and cell-wall biosynthesis, as well as a small GTPase that regulates nuclear membrane transport and mitotic processes in fungi. The cognate gene encoding the latter protein, designated *RanA*, was subsequently cloned and characterized. The *P. marneffei *RanA protein sequence, which contained the signature motif of Ran-GTPases, exhibited 90% homology to homologous *Aspergillus *proteins.

**Conclusion:**

This study clearly demonstrates the utility of proteomic approaches to studying dimorphism in *P. marneffei*. Moreover, this strategy complements and extends current genetic methodologies directed towards understanding the molecular mechanisms of phase transition. Finally, the documented increased levels of RanA expression suggest that cellular development in this fungus involves additional signaling mechanisms than have been previously described in *P. marneffei*.

## Background

The genus *Penicillium *is comprised of several hundred species of filamentous fungi (moulds) that are distributed world wide among quite diverse ecosystems [1-3]. The metabolic versatility of species within this taxon has had beneficial, as well as detrimental, impacts upon the environment and other living organisms [2,4-7]. Yet, prior to the 1980s, *Penicillium *species were generally considered medically insignificant having been reported to cause less than 80 cases of serious disease in humans since the late 1800s [8,9]. With the exception of a single species, the relative avirulent nature of monomorphic *Penicillium *species is reflected in that only four cases of infection have been noted in patients afflicted with AIDS [8,10,11]. The lone exception is *P. marneffei*, a highly significant pathogen of HIV-infected individuals living or having traveled in Southeast Asia [12,13].

In contrast to the monomorphic, vegetative life cycle typical of *Penicillium *species [14,15], *P. marneffei *expresses a second mode of cellular development. Like other Penicillia, *P. marneffei *grows at room temperature (25°C) as a multicellular mould. However, in vitro incubation of mould cultures at 37°C (body temperature) results in the production of yeast cells that divide by fission. These in vitro forms are virtually identical to the yeast cells of *P. marneffei *found in diseased tissue [16]. Furthermore, when the in vitro derived yeast cells are subsequently incubated at 25°C, the mould phase is regenerated. These observations confirm not only the dimorphic nature of *P. marneffei*, but also demonstrate that this conversion process is thermally regulated much like that of other fungal pathogens [17]. To date, *P. marneffei *represents the only known dimorphic species among the Penicillia [12,13].

**Figure 1:**
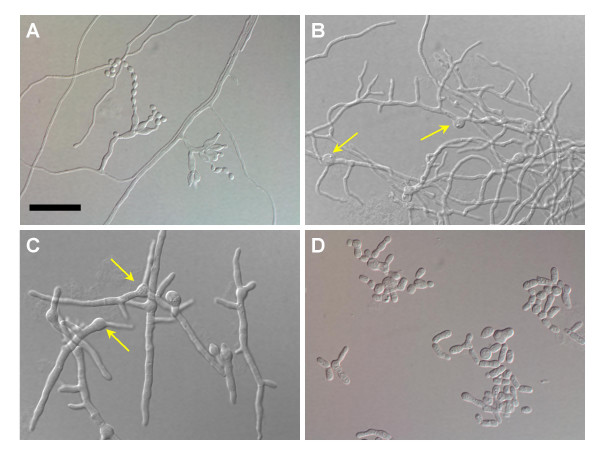
Thermal dimorphism in *P. marneffei*. A) The mould phase of *P. marneffei *depicting phialides bearing typical conidia (slide culture incubated at 25°C). B) Thin, multiply branched hyphae developing from conidia (arrows) incubated in SDB for 24 hours at 25°C. C) Short, broad hyphae generated from conidia (arrows) incubated in SDB for 24 hours at 37°C. D) Yeast cells of *P. marneffei *produced from conidia incubated in SDB for 96 hours at 37°C. The size bar in frame A, representing 20 μm, is applicable to each individual photomicrograph.

The dimorphic nature of *P. marneffei *has attracted investigators interested in not only understanding the underlying molecular mechanisms of its unique mode of cellular development, but also for the identification of novel targets that might be exploited in the development of new chemotherapeutic modalities. Initial studies were limited to the morphological features of clinical isolates and the cellular events in the transition of the mould phase to the yeast phase [18-20]. Subsequent morphological and genetic studies have refined the phenotypic observations of landmark events in this conversion process, better termed phase transition [8,16,21-23]. Like other *Penicillium *species, conidia (spores) of *P. marneffei *incubated in the proper environment swell prior to germination. During this germination period, the nucleus also is duplicated. The germling continues to grow apically producing true, septate filaments (hyphae) having multiple nuclei per cell. Continued incubation on solid media usually results in the development of the characteristic conidia-bearing structures of *Penicillium *species (Fig. 1A), whereas conidiation usually does not occur in shaken broth cultures. Instead, homogeneous cultures of hyphae are formed (Fig. 1B). By comparison, conidia incubated on solid or in liquid media at 37°C also form septate, multinucleate hyphae, but by 18–24 hours of incubation they appear wider and much shorter in length (Fig. 1C). As these determinate hyphae age, they fragment along septal planes generating reproductive spores termed arthroconidia. The arthroconidia continue to grow by briefly elongating prior to septum formation and fission, thereby forming yeast-like cells that continue to reproduce in the same manner (Fig. 1D). Eventually, such growth establishes a nearly homogeneous culture of yeast cells that are strikingly similar to those produced in vivo. Interestingly, both arthroconidia and the subsequently generated yeast cells are uninucleate. Significantly, reciprocal shifts in the incubation temperature of either the mould or yeast forms of *P. marneffei *entirely reverse the direction of the phase transition: uninucleate yeast cells transform into multinucleated hyphae, and vice versa. These observations suggest that the tightly controlled thermal regulation of phase transition in *P. marneffei *may be coupled with nuclear division [22,23].

The study of phase transition in *P. marneffei *has been aided by the establishment of molecular methodologies, many of which are similar to those previously used to study filamentous fungi like *Aspergillus *[22]. These methodologies have permitted the identification of potential diagnostic- and virulence-related genes, such as cell-wall antigens [24], catalase-peroxidase [25], heat-shock protein 70 [26], and isocitrate lyase [27], as well as a number of genetic factors related to intracellular signaling and polarized growth [12,22,23,28,29]. To date, no genes have been identified that specifically induce dimorphism in *P. marneffei*.

Despite the molecular advances in studying *P. marneffei*, rapid progress has been restricted in part due to the absence of a completely annotated and sequenced genome. Efforts are currently underway to remedy this situation (Alex Andrianopoulos, personal communication). Undoubtedly, the existence of a sequenced genome will be a tremendous asset in elucidating the molecular mechanisms of morphogenesis in *P. marneffei*. A complementary approach directed towards characterizing the *P. marneffei *proteome would augment such efforts, particularly investigations pertaining to those protein expression differences that occur during phase transition. Two recently published studies used peptide mass fingerprinting to identify a number of proteins that were differentially expressed during the later stages of mould and yeast development in *P. marneffei *[30,31]. In the present study, however, we report the results of our proteomic survey of the dimorphic process in *P. marneffei *that significantly differs in its approach to identifying proteins of interest.

Specifically, protein profiles in the present study were generated by two-dimensional gel electrophoresis (2DGE) from whole cell proteins extracted from the early stages of mould and yeast development in *P. marneffei*. Such profiles are more likely to reveal differences related to the molecular basis of phase transition than those used in previous studies that focused on fully differentiated cells [30,31]. Selected proteins were recovered, subjected to sequencing by mass spectrometry (MS), and putative identifications derived by searching available databases for homologous sequences in other fungi. The gene encoding one of these proteins was subsequently cloned and found to be a GTPase-like ortholog of the *Ran *gene from several fungal species, including *Aspergillus*. The collective results of this study not only demonstrate the utility of proteomic approaches to studying dimorphism in *P. marneffei*, but also that such a strategy complements and extends current genetic investigations.

## Results

### Experimental rationale

Cultures of *P. marneffei *incubated in Sabouraud dextrose broth at 25°C and 37°C exhibited distinct morphological differences within 24 hours that became pronounced upon further incubation (Fig. 1). At 25°C, conidia developed as multiply branched hyphae that were much thinner and longer than those produced at 37°C. The latter were much more broad and short. By 96 hours of incubation at 37°C, the hyphae converted to a yeast phase via arthroconidiogenesis (Fig. 1D). Both types of morphological development were replicated on solid media, the difference being that the mould phase of *P. marneffei *formed typical conidiophores bearing chains of ovoid conidia (Fig. 1A).

Since the differences in hyphal morphology represent a key developmental landmark that can be observed early in the phase transition of *P. marneffei*, we reasoned that the divergence in morphology should also be directly manifested in the protein profiles of these forms. Moreover, we further reasoned that the protein profiles at 24 hours of incubation would be more reflective of those molecular mechanisms involved in the induction of phase transition than those from later time points. Hence, the thrust of our proteomic analysis was focused on a single incubation time of 24 hours. Though we did examine some protein profiles of later stages of phase transition in *P. marneffei *(data not shown), we speculated that these patterns would be more indicative of pathways involved in maintenance and progression of morphological development rather than its initiation. Therefore, unless otherwise noted, the use of the terms "mould phase" and "yeast phase" in this report are employed to describe those data derived from 24-hour cultures incubated at 25°C and 37°C, respectively. Within the context of the present study, these terms are not meant to suggest the examination of the fully developed mould and yeast phenotypes.

To assess protein expression differences, our initial experiments studied protein profiles over the pI range of 3 – 10. However, we observed that a majority of proteins fell in the mid-pI ranges. Therefore, we focused the majority of our efforts to more effectively resolve and identify proteins in the pI range of 5 – 8.

### Overview of protein profile differences

**Figure 2:**
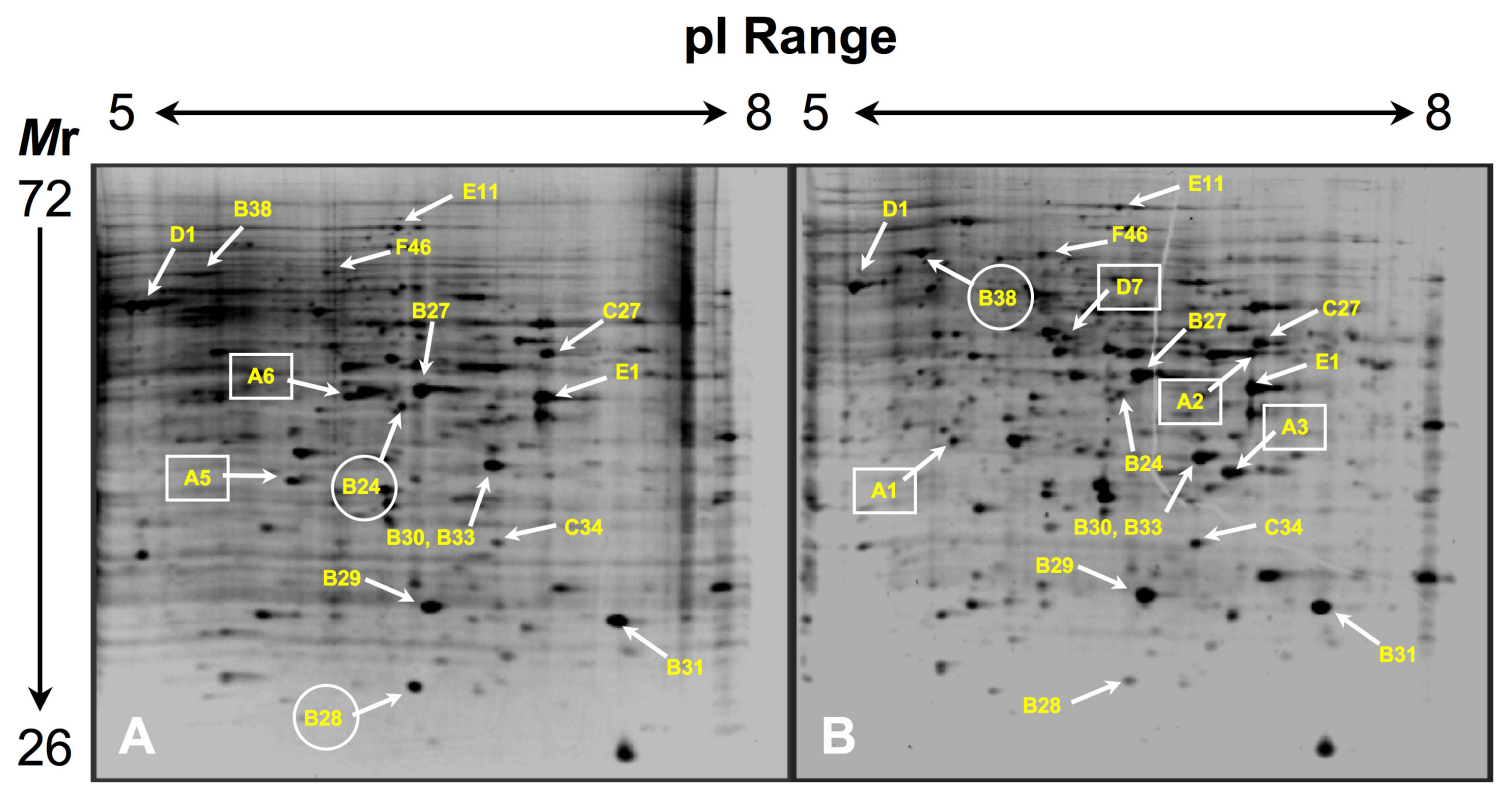
Negative image of SYPRO Ruby stained gels depicting proteins resolved by 2DGE over a 5 – 8 pI range. The protein profile was generated from cell extracts of conidia incubated in SDB for 24 hours at 25°C (A) or 37°C (B). Labeled proteins correspond to those listed in Tables 1-3). Those labels that are boxed denote up-regulated proteins that are essentially unique to the mould or yeast phase. Those labels enclosed within a circle mark proteins expressed at higher levels in a particular growth form than its counter part in the companion culture. The approximate pI and molecular mass ranges of the gel are shown at the top and to the left of these figures.

**Figure 3:**
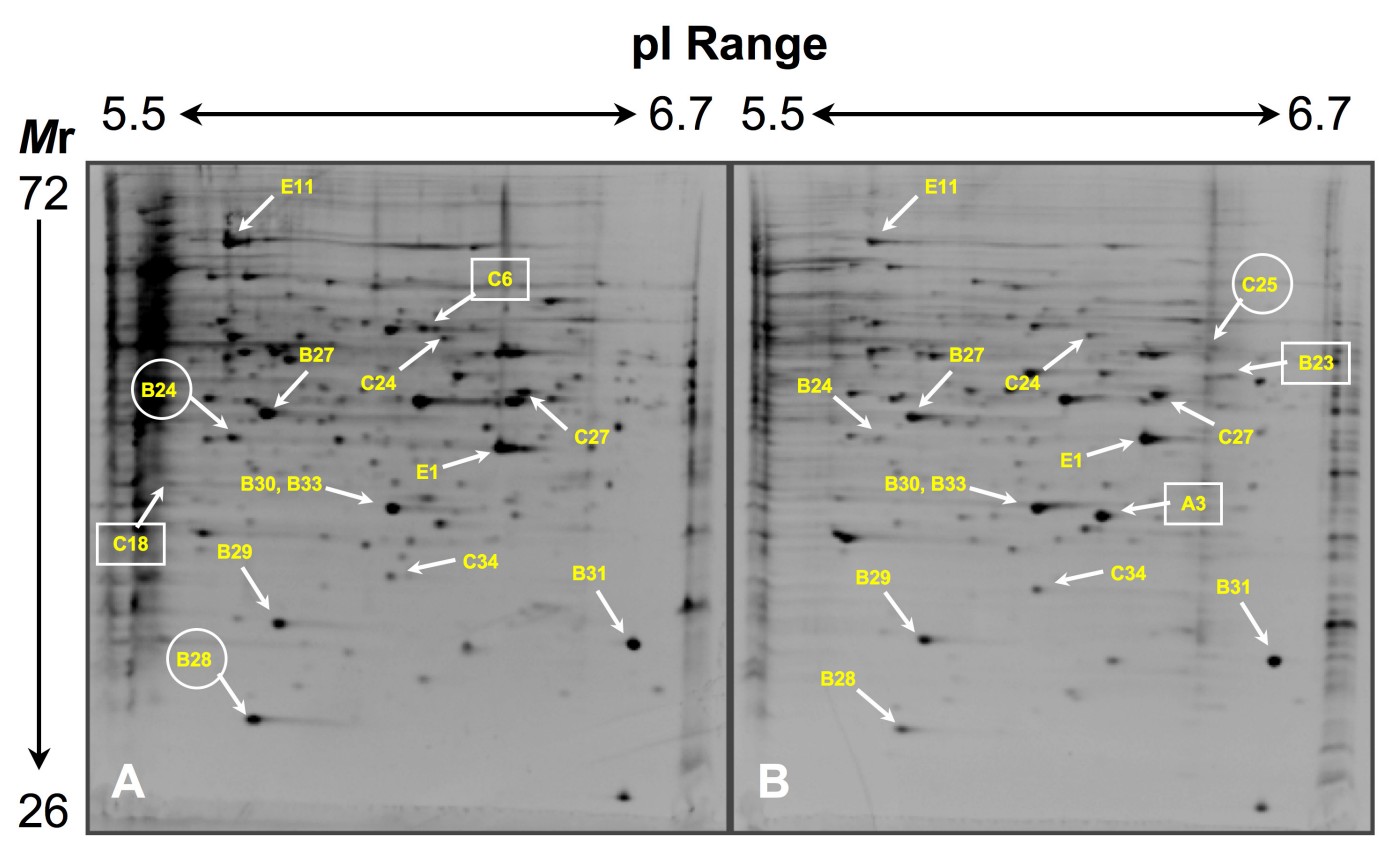
Negative image of SYPRO Ruby stained gels depicting protein resolved by 2DGE over a 5.5 – 6.7 pI range. Proteins were resolved from cell extracts of conidia incubated in SDB for 24 hours at 25°C (A) or 37°C (B). Labeled proteins correspond to those listed in Tables 1-3. Those labels that are boxed denote up-regulated proteins that are essentially unique to the mould or yeast phase. Those labels enclosed within a circle mark proteins expressed at higher levels in a particular growth form than its counter part in the companion culture. The approximate pI and molecular mass ranges of the gel are shown at the top and to the left of these figures.

**Figure 4:**
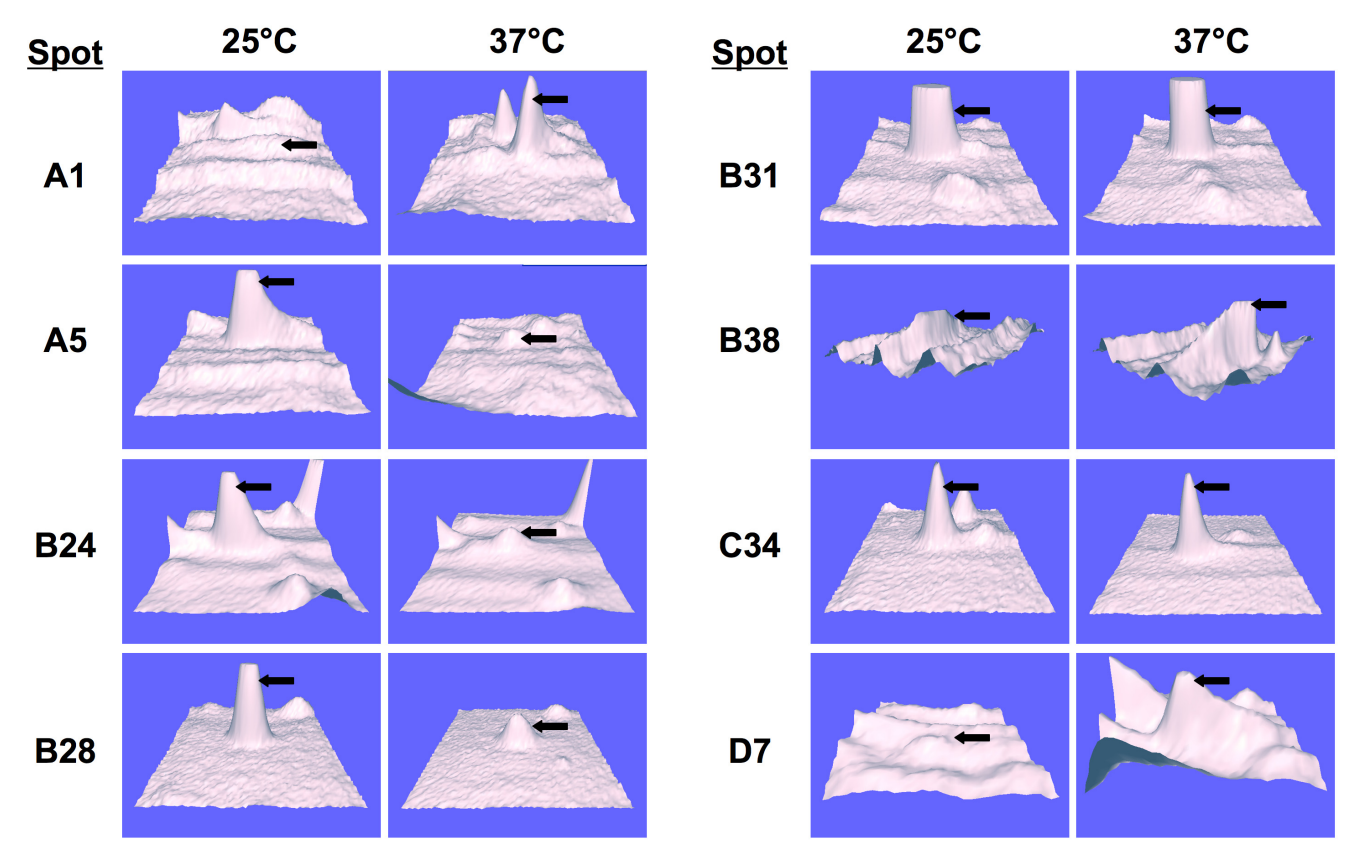
Expression levels of selected *P. marneffei *proteins resolved by 2DGE. These three-dimensional contour maps document selected examples of proteins preferentially expressed at 37°C (spots A1, B38, and D7) or at 25°C (spots A5, B24, and B28), as well as proteins that are expressed at approximately equivalent levels at these incubation temperatures (spots B31 and C34). The arrows in each diagram denote the protein peak being measured. For comparison, see Fig. 2.

Representative 2DGE results are depicted in Figures 2 and 3. On average, analysis using the PDQuest Software system revealed 270 detectable spots per gel from each match set of protein profiles representing pI ranges of 5–8 and 5.5–6.7. To assess the expression levels of a given protein, the staining intensities of spatially paired spots between parallel gels of the mould and yeast phases were compared. Spots were ascribed as representing either a "common" protein or one of two types of differentially expressed proteins. A protein was defined as "common" if consistently near-equal levels of expression, as determined by spot intensity in each experimental replicate, were observed in gels prepared from yeast- and mould-phase extracts (e.g., spot B31; Figs. 2, 3, 4). In contrast, a spot from such gels was considered to denote an up-regulated, differentially expressed protein if its staining intensity was consistently higher than its corresponding companion spot in the opposite growth form. For example, spots B24 and B28 (Figs. 2, 3, 4) are considered up-regulated "mould-phase" proteins. A second type of differentially expressed protein included those determined to be unique to a specific growth form. Such a protein was defined as one not having a spatially matched spot in parallel gels, i.e., expression in the opposite growth form was below the detection limit of the SYPRO Ruby stain. For example, spot A1 (Figs. 2 and 4) represents a unique "yeast-phase" protein not having an observable counterpart in the mould-phase gel.

From our collective experiments, a total of 45 spots were selected from gels of various pI ranges for MS sequencing. Because no completely annotated and sequenced genome of *P. marneffei *has been released, the putative identifications and functions of all these spots were determined by searching for homologies among fungal proteins within available databases. The results of these analyses are described within the text below as well as presented in Tables 1, 2, 3. Each peptide listed in the tables possessed ion scores above the lowest acceptable values (typically ranging from 44 to 56) indicating identity or extensive homology (p < 0.05). The individual proteins within each of these groups differed in experimentally derived pI, mass, or both. Finally, throughout these investigations, we noted that on occasion a small number of protein identities would change due to the expanding database. The peptide identifications noted in Tables 1, 2, 3 and in the text, however, reflect a final updated database search that was performed in April 2008. From the spots analyzed, putative identifications based upon at least two peptide matches were assigned to 15 common proteins, 4 mould-phase proteins, and 12 yeast-phase proteins. Tentative identifications based upon a single peptide match were made for 2 common proteins, 7 mould-phase proteins, and 5 yeast-phase proteins. Among all spots examined, 5 from each of the mould and yeast phases were determined to be unique.

**Table 1:** Identification of proteins expressed at equivalent levels in developing yeast and mould phases of *P. marneffei*.^a^

**Spot (Fig.)**	**Protein Name**	**NCBI Accession (Version)**	**Theo. pI/*M*r (kDa)**	**Expt. pI/*M*r (kDa)**	**Species**	**Mascot Score**	**NP/PD**	**MS/MS Peptide Sequence**	**SC (%)**
B27 (2, 3)	hypothetical protein (malate dehydrogenase)	XP_001817504 (gi|169766066)	8.79/35.7	5.8/52.6	*Aspergillus oryzae*	2597	9/122	R.VSELALYDIR.G K.RLFGVTTLDVVR.A R.LFGVTTLDVVR.A R.MAESLLK.A + OM R.IQFGGDEVVK.A R.DDLFNTNASIVR.D K.DGAGSATLSMAMAGAR.M + 2 OM K.AKDGAGSATLSMAMAGAR.F + 2 OM K.VELGPNGVEKILPVGQVNAYEEK.L	27
B29 (2, 3)	Cu,Zn-superoxide dismutase	ABD67502 (gi|89329757)	5.74/16.1	5.9/35.0	*Penicillium marneffei*	5183	9/139	M.VKAVAVLR.G K.GTVTFEQADENSPTTISWNITGHDANAER.G K.THGAPTDDER.H K.THGAPTDDERHVGDLGNFK.T R.HVGDLGNFK.T K.TDAQGNAVGFVEDK.L K.LIGAESVLGR.T R.TIVVHAGTDDLGR.G K.TGNAGPRPACGVIGISA.-	71
B30 (2, 3)	hypothetical protein (peroxiredoxin)	XP_001226164 (gi|116200704)	5.57/25.1	6.1/45.9	*Chaetomium globosum*	202	2/25	R.VVKPYLR.F R.TILSYPASTGR.N K.IAYLYDMIDYQDTTNVDEK.G + OM R.KIAYLYDMIDYQDTTNVDEK.G + OM	16
B31 (2, 3)	hypothetical protein (nucleotide diphosphate kinase)	XP_001393898 (gi|145242650)	8.45/17.2	6.5/33.9	*Aspergillus niger*	413	5/127	R.GLVGPIISR.F R.TILGATNPLASAPGTIR.G R.GDYAIDVGR.N R.NVCHGSDSVENAQK.E K.HSQFDWIYEK.A	38
B33 (2, 3)	hypothetical protein (malate dehydrogenase)	XP_001246025 (gi|119190837)	9.19/36.0	6.1/45.9	*Coccidioides immitis*	328	5/9	R.FAESLLK.A R.IQFGGDEVVK.A R.VSQLALYDIR.G R.LFGVTTLDVVR.A R.DDLFNTNASIVR.D	14
B37 (-)	ATP synthase F1, beta subunit	XP_753589 (gi|70997705)	5.30/55.6	4.5/32.5	*Aspergillus fumigatus*	219	3/3	K.AHGGYSVFTGVGER.T R.EGNDLYHEMQETGVIQLEGESK.V + OM K.SLQDIIAILGMDELSEADKLTVER.A + OM	11
C27 (2, 3)	hypothetical protein (ketol-acid reducto-isomerase)	XP_363882 (gi|39952331)	8.22/44.9	6.3/54.9	*Magnaporthe grisea*	154	2/4	R.DNGLNVIIGVR.K K.DDTLALIGYGSQGHGQGLNLR.D	8
C34 (2, 3)	peptidyl-prolyl cis-trans isomerase B precursor (cyclophilin)	XP_001728304 (gi|1164426123)	8.61/22.5	6.1/40.0	*Neurospora crassa*	69	3/4	R.IVMGLYGK.T + OM K.GLLSMANAGK.D + OM K.HVVFGEVLEGYDVVEK.I	16
D1 (2)	ATP synthase F1, subunit beta	XP_753589 (gi|70997705)	5.30/55.6	5.3/58.9	*Aspergillus fumigatus*	8540	22/277	K.LVDLK.D K.IGLFGGAGVGK.T K.LVDLKDTIR.S K.VVDLLAPYAR.G R.VQQMLQEYK.S + OM K.IHQVIGAVVDVK.F R.TIAMDGTEGLTR.G + OM R.IVNVTGDPIDER.G R.FTQAGSEVSALLGR.I K.AHGGYSVFTGVGER.T R.VALTGLTIAEYFR.D K.TVFIQELINNIAK.A K.VALVFGQMNEPPGAR.A + OM R.GISELGIYPAVDPLDSK.S R.DEEGQDVLLFIDNIFR.F R.FLSQPFTVAQVFTGIEGK.L K.SLQDIIAILGMDELSEADK.L R.EGNDLYHEMQETGVIQLEGESK.V + OM K.SLQDIIAILGMDELSEADKLTVER.A + OM K.AIINGEGDDLPEAAFYMVGDFESAR.A + OM R.TREGNDLYHEMQETGVIQLEGESK.V + OM K.GSITSVQAVYVPADDLTDPAPATTFAHLDATTVLSR.G	58
D11 (-)	3-isopropyl-malate dehydrogenase	XP_752944 (gi|70996378)	5.32/39.3	5.5/47.0	*Aspergillus fumigatus*	323	5/13	R.NVIEAGIR.T R.GVDFNIIR.E R.ELTGGIYFGDR.K K.NADAVLLGAIGGPK.W K.VNGIYEPIHGSAPDIAGK.G	16
E1 (2, 3)	RACK1-like protein	ABA33785 (gi|75993570)	6.59/35.5	6.3/51.3	*Para-coccidioides brasiliensis*	271	7/13	K.FTITDK.G R.LWELATGNTTR.T K.VDELKPEYVEK.G K.DGTTMLWDLNESK.H + OM K.SKVDELKPEYVEK.G R.FSPNPQNPVIVSAGWDK.L R.IQTDHIGHTGYINTVTISPDGSLCASGGK.D	28
E11 (2, 3)	hypothetical protein (aconitase)	XP_001393157 (gi|145241021)	6.18/84.9	5.7/66.2	*Aspergillus niger*	536	10/21	K.DIILK.V K.FNPLTDK.L K.GNYINYKK.M R.HLGGLAIITR.S K.FNPLTDKLK.D K.SLFTVTPGSEQIR.A K.EVYDFLASACAK.Y K.KGEANSIVSSYNR.N K.VGLIGSCTNSSYEDMSR.G + OM R.GHLDNISNNMLIGAINEANGEANKVK.N + OM	11
E19 (-)	glyceraldehyde 3-phosphate dehydrogenase	ABV02551 (gi|157057025)	7.01/36.6	7.1/50.0	*Penicillium marneffei*	818	12/31	K.VGINGFGR.I K.YDTQHGQFK.G K.GTIEVEGSDLIVNGK.R K.FYQERDPANIK.W K.SSDTIISNASCTTNCLAPLAK.I R.TAAQNIIPSSTGAAK.A K.VIPALNGK.L K.LTGMSMRVPTSNVSVVDLTCR.T R.TEKPVSYDEIK.A K.KYAEGELK.G K.GIMGYTEDDVVSTDMNGNSNSSVFDAK.A + OM K.AGIALNSNFIK.L	48
E26 (-)	60s ribosomal protein L2	XP_362607 (gi|39946140)	10.89/40.0	6.8/54.0	*Magnaporthe grisea*	132	3/3	K.DFLLPSNVVSQADLTR.L R.LINSSEIQSVLR.A R.LINSSEIQSVLRAPKGTAR.T	9
F46 (2)	enolase	Q76KF9 (gi|74662366)	5.14/47.3	6.0/58.1	*Penicillium chrysogenum*	850	11/35	K.LNQILR.I K.VDEFLNK.L K.SCNALLLK.V K.KYDLDFK.N K.IAMDVASSEFYK.E + OM K.GVPLYAHISDLAGTK.K K.WLTYEQLADLYK.S K.VNQIGTLTESIQAAK.D R.GNPTVEVDVVTETGLHR.A K.TSDFQIVGDDLTVTNPLR.I R.LAFQEFMIVPDTAPTFSEGLR.Q + OM	31

^a ^Spot, spot number corresponds to labeled spots in Figs. 2 and 3 (as indicated in parentheses); Protein name, matched protein description; NCBI Accession (Version), accession number and submission version of matched protein from NCBI database; Theo. pI/*M*r (kDa), theoretical isoelectric point and molecular mass based on amino acid sequence of the identified protein; Expt. pI/*M*r (kDa), experimental isoelectric point and molecular mass estimated from the 2DGE gels; Species, the fungal species of the matched protein; Mascot score, score obtained from the Mascot search for each match; NP, the number of matched peptides; PD, the number of peptides detected; MS/MS Peptide Sequence, amino acid sequences of peptides identified by Nano-LC/MS/MS; OM, oxidized methionine residue; SC, percent amino acid sequence coverage for the identified protein.

**Table 2:** Identification of preferentially expressed proteins in the developing mould phase of *P. marneffei*.^a^

**Spot (Fig.)**	**Protein Name**	**NCBI Accession (Version)**	**Theo. pI/*M*r (kDa)**	**Expt. pI/*M*r (kDa)**	**Species**	**Mascot Score**	**NP/PD**	**MS/MS Peptide Sequence**	**SC (%)**
B28 (2, 3)	hypothetical protein (malate dehydrogenase, mitochondrial precursor)	XP_001817504 (gi|169766066)	8.79/35.7	5.8/28.0	*Aspergillus oryzae*	591	8/12	R.VSELALYDIR.G R.IQFGGDEVVK.A R.LFGVTTLDVVR.A R.DDLFNTNASIVR.D K.RLFGVTTLDVVR.A K.DGAGSATLSMAMAGAR.M + 2 OM K.AKDGAGSATLSMAMAGAR.M + 2 OM K.VELGPNGVEKILPVGQVNAYEEK.L	25
C7 (-)	hypothetical protein (S-adenosyl-L-homocysteine hydrolase)	XP_658867 (gi|67521612)	6.02/49.3	5.7/60.5	*Aspergillus nidulans*	161	4/5	R.ATDVMIAGK.V + OM K.VAVVAGFGDVGK.G K.VADISLAAFGR.R K.SVQNIKPQVDR.Y	9
C18 (3)	proteasome component Pre9	XP_750785 (gi|70991873)	5.80/28.5	5.7/49.1	*Aspergillus fumigatus*	165	4/5	K.IEFATVGK.T R.TTIFSPEGR.L K.LLEQDTSAEK.L K.LSSEKIEFATVGK.T	12
F47 (-)	enolase	AAK51201 (gi|13991101)	5.33/47.3	6.0/37.0	*Penicillium citrinum*	105	2/2	K.VNQIGTLTESIQAAK.D R.LAFQEFMIVPDTAESFSEGLR.Q	8

^a ^Spot, spot number corresponds to labeled spots in Figs. 2 and 3 (as indicated in parentheses); Protein name, matched protein description; NCBI Accession (Version), accession number and submission version of matched protein from NCBI database; Theo. pI/*M*r (kDa), theoretical isoelectric point and molecular mass based on amino acid sequence of the identified protein; Expt. pI/*M*r (kDa), experimental isoelectric point and molecular mass estimated from the 2DGE gels; Species, the fungal species of the matched protein; Mascot score, score obtained from the Mascot search for each match; NP, the number of matched peptides; PD, the number of peptides detected; MS/MS Peptide Sequence, amino acid sequences of peptides identified by Nano-LC/MS/MS; OM, oxidized methionine residue; SC, percent amino acid sequence coverage for the identified protein.

**Table 3:** Identification of preferentially expressed proteins in the developing yeast phase of *P. marneffei*.^a^

**Spot (Fig.)**	**Protein Name**	**NCBI Accession (Version)**	**Theo. pI/*M*r (kDa)**	**Expt. pI/*M*r (kDa)**	**Species**	**Mascot Score**	**NP/PD**	**MS/MS Peptide Sequence**	**SC (%)**
A1 (2)	Ran GTPase spi1	NP_596827 (gi|19113619)	7.00/24.8	5.7/45.9	*Schizosaccharomyces pombe*	173	3/3	R.HLTGEFEK.K K.LVLVGDGGTGK.T K.NLQYYDISAK.S	13
A2 (2)	succinyl-CoA synthetase alpha subunit	DQ666366 (gi|110227027)	5.88/27.7	7.0/54.4	*Penicillium marneffei*	321	5/5	K.TLRDEFVR.R R.MGHAGAIVSGGK.G + OM K.IGIMPGFIHK.R + OM K.ISALEAAGVIVER.S K.AGTTHLDLPVFATVSDAVK.E	23
A3 (2,3)	heat shock protein 30	ABF82266 (gi|106647229)	6.17/20.7	6.2/45.0	*Penicillium marneffei*	752	9/31	R.SGDFAPLFR.L R.LLDDYDLHR.S R.DGQTPASSSISSFAPR.F K.DAYHLDGELPGIAQK.D K.DVEIEFSDPQTLTIK.G R.EYHTLPENENPHAPKPASVEDAPESSDETAVQ K.S R.SVGEFHR.S K.ASLKNGVLTVTVPK.A K.NGVLTVTVPK.A	66
B23 (3)	NAD-dependent formate dehydrogenase	XP_001270055 (gi|121703582)	7.65/46.2	6.5/57.4	*Aspergillus clavatus*	181	3/26	K.NEYDLENK.V K.VVGTVAVGR.I K.VLMVLYDGGEHAK.Q + OM	7
B38 (2)	antigenic mitochondrial protein HSP60	EAL93225 (gi|71001164)	5.53/62.1	5.6/61.8	*Aspergillus fumigatus*	1000	13/57	K.FENLGAR.L K.LSGGVAVIK.V R.LLQDVASK.T K.AVTSTLGPK.G R.AIFSETVK.N R.GQLQVAAVK.A R.VVDALNATR.A K.APGFGDNRK.S K.VGGASEVEVGEK.K K.EDTIILNGEGSK.D K.NVAAGCNPMDLR.R + OM K.VEFEKPLILLSEK.K R.GVMADPSTSEYEKEK.L + OM	22
B40 (-)	ATP synthase F1, subunit beta	XP_753589 (gi|70997705)	5.30/55.6	4.2/47.0	*Aspergillus fumigatus*	1047	12/17	K.IGLFGGAGVGK.T K.VVDLLAPYAR.G K.IHQVIGAVVDVK.F R.FTQAGSEVSALLGR.I K.AHGGYSVFTGVGER.T K.TVFIQELINNIAK.A K.VALVFGQMNEPPGAR.A + OM R.GISELGIYPAVDPLDSK.S R.FLSQPFTVAQVFTGIEGK.L R.EGNDLYHEMQETGVIQLEGESK.V + OM K.SLQDIIAILGMDELSEADKLTVER.A + OM K.GSITSVQAVYVPADDLTDPAPATTFAHLDATTVLSR.G	39
C25 (3)	Phospho-glycerate kinase	XP_752401 (gi|70995281)	6.31/44.8	6.4/59.7	*Aspergillus fumigatus*	391	8/11	K.TVGDIIEK.A R.IVGALPTIK.Y K.IQLIDNLLPK.V K.ASGGQVILLENLR.F R.AHSSMVGVDLPQK.A + OM K.YSLKPVVPELEK.L K.VSDKIQLIDNLLPK.V K.LSHVSTGGGASLELLEGK.E	20
D7 (2)	hypothetical protein (activator of HSP90 ATPase)	EAS36198 (gi|90306567)	5.11/36.5	6.1/56.1	*Coccidioides immitis*	164	2/7	K.IVSMDGDVDVSQR.K + OM R.TTAEEMYTTFTDPQR.L	8
D15 (-)	cell division control protein Cdc48	XP_756045 (gi|71002728)	5.09/90.5	5.1/67.4	*Aspergillus fumigatus*	221	3/3	R.EVDIGIPDPTGR.L R.VVSQLLTLMDGMK.A + 2 OM R.VVNQLLTEMDGMTSK.K + 2 OM	4
D16 (-)	heat shock protein 70	AAX63812 (gi|62125797)	5.03/69.6	5.1/64.0	*Penicillium marneffei*	1544	21/36	R.TKDNNLLGK.F R.LSKEEIER.M K.EKLEAEIEK.T R.LVNHFASEFK.R K.FELTGIPPAPR.G K.FSDPEVQADAK.H K.DAGLIAGLNVLR.I R.IEIIANDQGNR.T K.YKAEDEAEATR.I R.KFSDPEVQADAK.H K.SSVHEIVLVGGSTR.I K.LVTDFFNKEPNK.S R.TTPSFVAFTDSER.L R.EADEVEERPEELD.- R.DDRIEIIANDQGNR.T K.QFTPEEISSMVLIK.M + OM K.ATAGDTHLGGEDFDSR.L K.NQVAMNPHNTVFDAK.R + OM R.IINEPTAAAIAYGLDKK.V K.SINPDEAVAYGAAVQAAILSGDTSSK.S R.GVPQIEVTFDMDANGIMNVSAVEK.G + 2 OM	40
D18 (-)	hypothetical protein (UDP-N-acetylglucosamine pyrophos-phorylase)	XP_682363 (gi|67904214)	6.03/56.0	5.9/61.2	*Aspergillus nidulans*	160	3/3	K.SLFQLQAER.I R.REDEFSPLK.N R.NATESVGLILQK.N	5
E7 (-)	adenylate kinase	XP_750499 (gi|70991300)	7.71/28.9	6.3/44.8	*Aspergillus fumigatus*	206	3/6	K.SIAEQMR.I + OM K.NGFILDGFPR.T K.IMDQGGLVSDEIMVNMIK.N + 3 OM	13

^a ^Spot, spot number corresponds to labeled spots in Figs. 2 and 3 (as indicated in parentheses); Protein name, matched protein description; NCBI Accession (Version), accession number and submission version of matched protein from NCBI database; Theo. pI/*M*r (kDa), theoretical isoelectric point and molecular mass based on amino acid sequence of the identified protein; Expt. pI/*M*r (kDa), experimental isoelectric point and molecular mass estimated from the 2DGE gels; Species, the fungal species of the matched protein; Mascot score, score obtained from the Mascot search for each match; NP, the number of matched peptides; PD, the number of peptides detected; MS/MS Peptide Sequence, amino acid sequences of peptides identified by Nano-LC/MS/MS; OM, oxidized methionine residue; SC, percent amino acid sequence coverage for the identified protein.

### Common mould- and yeast-phase proteins

Fifteen proteins common to both developing yeast and mould phases of *P. marneffei *were identified by at least two peptides sequenced by MS (see Table 1; Figs. 2 and 3). The sequence coverage for each common protein ranged from 8 to 71% of the identified ortholog with concurrent Mascot scores ranging from 69 to 8540. The putative functions identified for these proteins can be divided into several groups: cellular protection from oxygen radicals (Cu, Zn-superoxide dismutase [spot B29]); general metabolism and energy production (ATP synthase [spots B37 and D1]; 3-isopropylmalate dehydrogenase [spot D11]; glyceraldehyde 3-phosphate dehydrogenase [spot E19]; and enolase [spot F46]); signal transduction (cyclophilin [spot C34] and RACK1-like protein [spot E1]); ribosomal structure (60S ribosomal protein L2 [spot E26]); and six proteins identified as hypothetical. Of these six hypothetical proteins, each bore apparent homology to a known protein involved either in antioxidant activity (peroxiredoxin [spot B30]), metabolism (malate dehydrogenase [spots B27 and B33]; nucleotide diphosphate kinase [spot B31]; and aconitase [spot E11]), or amino acid transport (ketol-acid reductoisomerase; spot C27). Two other putative identities of common yeast- and mould-phase proteins were derived based upon a single peptide sequence (data not shown). These proteins include fumarase and a protein of unknown function seemingly related to the CipC protein of *Emerciella nidulans *(teleomorph of *A. nidulans*; NCBI Accession No. CAC87272).

Several important observations were made from these data. First, many of the above-noted proteins were identified in different gels varying in acrylamide concentration and prepared over various pI ranges. For example, an ortholog of spot C27, a putative ketol-acid reductoisomerase from *Magnaporthe grisea*, was identified in an 8% gel at a different spatially relative location, but having sequence homology and equivalent mass/pI values to the same enzyme in *Neurospora crassa *(data not shown). Second, the extracted proteins resolved in the same relative spatial relationship to one another. For example, Figures 2 and 3 attest that there was no disparity in the relative positions of spots containing peroxiredoxin (spot B30), malate dehydrogenase (spots B27 And B28), Cu,Zn-superoxide dismutase (spot B29), RACK1 (spot E1), cyclophilin (spot C34), and several hypothetical proteins. Similar observations were made of specific spots migrating to a spatially relative position in gels having distinct pI ranges of 3–6, 4.7–5.9, and 6.3–8.3 (data not shown). Collectively, these two observations provide evidence supporting the reproducibility of our protocols. Third, MS sequencing was able to identify two distinct proteins, peroxiredoxin and malate dehydrogenase (spots B30 and B33, respectively), which resolved as a single spot (Figs. 2 and 3). Fourth, a pair of spots containing malate dehydrogenase (spots B27 and B33) were identified from different locations within in the same 2DGE gels, i.e., having different experimentally derived pI and/or mass values (Figs. 2 and 3; see Table 1). A similar situation was observed for ATP synthase (see Table 1). In both of these cases, functionally identical proteins were identified among proteins preferentially expressed by either the mould or yeast phases of *P. marneffei *(see spot B28, Table 2; spot B40, Table 3). Finally, all the proteins identified as common to both the mould and yeast phases were determined to be homologous to fungal proteins, including two *P. marneffei *homologs (Cu,Zn-superoxide dismutase [spot B29] and glyceraldehyde 3-phosphate dehydrogenase [spot E19]) and an enolase (spot F46) from the related species, *P. chrysogenum*.

### Mould-phase proteins of *P. marneffei*

Among the mould-phase proteins examined, four were identified by two or more MS-generated sequences (see Table 2; Fig. 2, 3, 4). The sequence coverage for each mould-phase protein ranged from 8 to 25% of the identified ortholog with concurrent Mascot scores ranging from 105 to 591. One of these proteins, an ortholog of a proteosome component (spot C18; Fig. 3A) seemed to be expressed solely during development of the mould phase. The three remaining proteins appeared up-regulated over the levels expressed in the yeast phase. These proteins included enolase (spot F47), a hypothetical protein encoding malate dehydrogenase (spot B28; Figs. 2, 3, 4), and a hypothetical protein that shares homology with S-adenosyl-L-homocysteine hydrolase (spot C7).

Seven additional mould-phase proteins that consistently appeared as spots in 2DGE gels were tentatively identified based upon a single peptide sequence (data not shown). Four of these appeared to be unique including an unknown predicted protein from *Chaeotomium globosum *(spot A5; Fig. 2A and 4), a ribosomal structural protein (spot A6; Fig. 2A), dihydrolipimide dehydrogenase (spot C6; Fig. 3A), and a hypothetical protein that appears homologous to an activator of the heat-shock protein 90 (HSP90) ATPase. Three other up-regulated proteins were identified as a urease accessory protein (spot B24; Figs. 2, 3, 4), an orinithine transaminase, and a translation elongation factor. Again, all of these proteins were identified based upon homology to fungal proteins, including the related species, *P. citrinum*.

### Yeast-phase proteins of *P. marneffei*

12 proteins that appeared to be preferentially expressed in the developing yeast phase of *P. marneffei *were identified by two or more peptides sequenced by MS (see Table 3; Fig. 2, 3, 4). Seven appeared to be up-regulated over levels of the same protein expressed in the mould phase, whereas 5 others (spots A1, A2, A3, B23, and D7) seemed to be uniquely expressed by the yeast phase of *P. marneffei*. The Mascot scores for these identified proteins ranged from 160 to 1544 with concurrent sequence coverage for each protein ranging from 4 to 66% of the identified ortholog. As expected, some of these proteins apparently function in a heat-shock response (spots A3 [Figs. 2 and 3]; B38 [Figs. 2 and 4]; and D16). Two of these proteins were identified from *P. marneffei*, including heat shock protein 70 in which the cognate gene was recently cloned [26]. Furthermore, a hypothetical protein (spot D7; Figs. 2 and 4) identified in the analysis of the developing *P. marneffei *yeast phase exhibited significant homology to an activator of HSP90 ATPase from *Coccidioides immitis*. Many of the remaining proteins had homology to those that function in energy production and other cellular metabolic pathways (see Table 3). These proteins included an NAD-dependent formate dehydrogenase (spot B23; Fig. 3B), the beta subunit of ATP synthase (spot B40), adenylate kinase (spot E7), phosphoglycerate kinase (spot C25; Fig. 3B), and the *P. marneffei *homolog of a subunit of succinyl-CoA synthetase (spot A2; Fig. 2B).

The remaining three yeast-phase proteins represented orthologs with very different functions than any of the other proteins identified in this study. One is homologous to Cdc48p from *A. fumigatus *(spot D15), another to UDP-N-acetylglucosamine pyrophosphorylase (spot D18), and the remaining one to the spi1 protein of *Schizzosaccharomyces pombe *(spot A1; Figs. 2 and 4). Subsequent investigations, described below, showed that this latter protein is more closely related to the spi1 orthologs, designated RanA, in several species of *Aspergillus*.

Five additional yeast-phase proteins were tentatively identified based upon a single peptide sequence (data not shown). These proteins consistently appeared as spots in 2DGE gels and included succinate dehydrogenase, three isoforms exhibiting homology to a bacterial heat shock protein, and another hypothetical protein exhibiting homology to an activator of HSP90 ATPase from *A. nidulans*. Interestingly, the same *A. nidulans *ortholog was identified as an up-regulated mould-phase protein, but having different pI and mass values (data not shown).

### Isolation of the *P. marneffei RanA *gene

**Figure 5:**
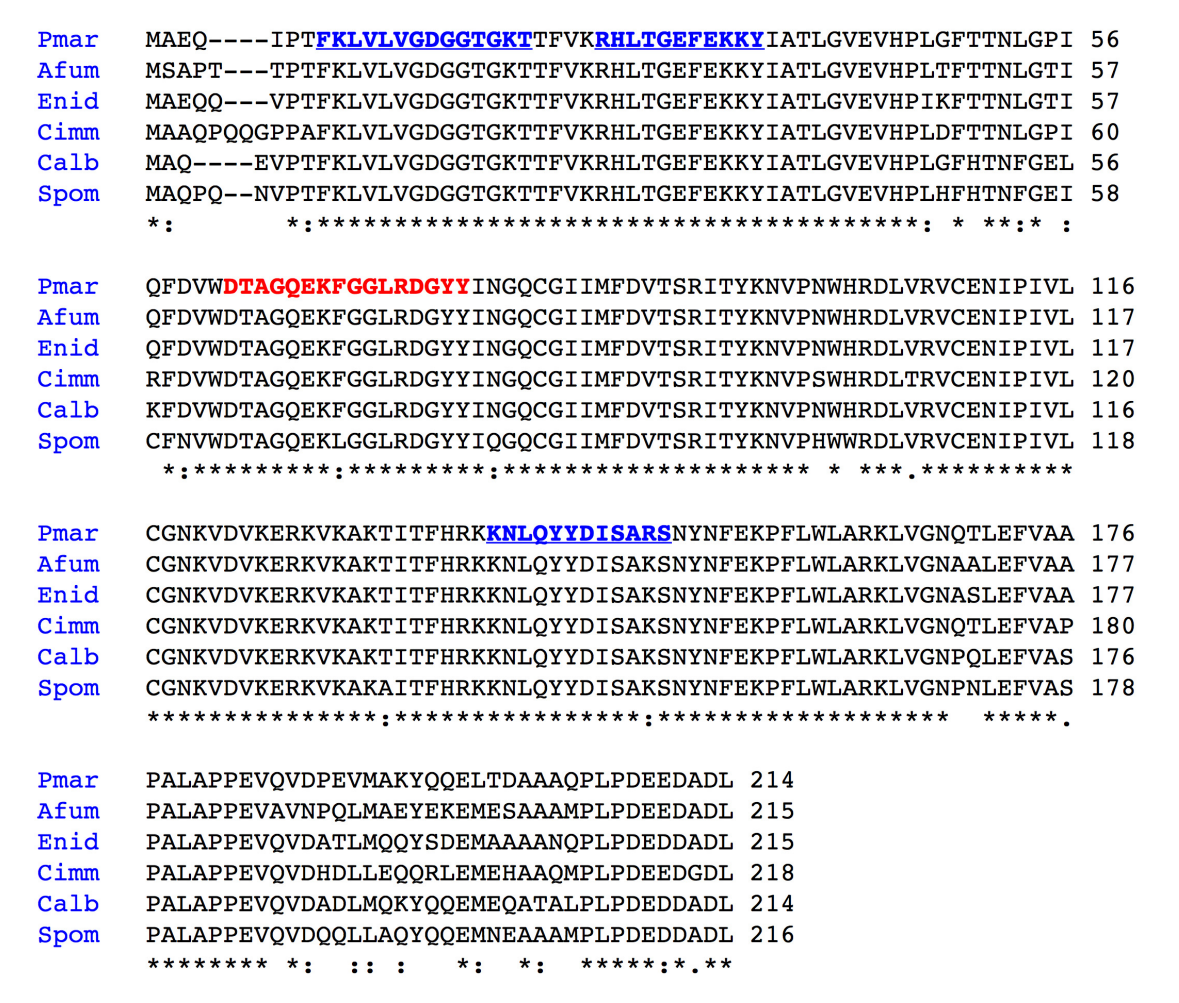
Alignment of amino acid sequences from RanA-like fungal proteins. The peptide sequences depicted include those from *P. marneffei *(Pmar; this study), *Aspergillus fumigatus *(Afum; NCBI Accession No. XP_751206), *E. nidulans *(Enid; NCBI Accession No. AAR08135), *Coccidioides immitis *(Cimm; NCBI Accession No. XP_001239221), *Candida albicans *(Calb; NCBI Accession No. XP_711509), and *S. pombe *(Spom; NCBI Accession No. NP_596827). Shared amino acid residues are indicated by a "*", whereas conserved and semi-conserved substitutions are noted by ":" and ".", respectively. The segment of amino acids in bold red font represents the Ran signature motif. In addition, the underlined amino acids in bold blue font denote those peptides originally identified by MS sequencing (see Table 3). Primer RAN-1F was designed to the nucleotide sequence encoding the C terminal end of the first underlined peptide, whereas RAN-1R was designed to the nucleotide sequence encoding the N terminal end of the third underlined peptide.

Based upon the MS-generated peptide sequences for the apparent RanA (spi1) ortholog of *P. marneffei *(spot A1, see Table 3), degenerate primers were designed for use in a PCR with genomic DNA. The resulting amplification product was 774 bp in size. This fragment was cloned, sequenced, then subjected to a homology search of nucleotide databases that showed it to be highly similar to a number of fungal small GTPases (data not shown). Using RACE and DNA walking procedures, this sequence was then extended to 1525 bp that contained the entire putative *RanA *gene of *P. marneffei *in addition to small segments of upstream and downstream sequences. Within *P. marneffei RanA *gene, four introns were identified based upon canonical splice signal sequences [32,33]. The sites of these four introns were subsequently confirmed by sequencing a cDNA copy of the gene (data not shown). When the deduced amino acid sequence (Fig. 5) was subjected to a BLAST search of GenBank, the results revealed a highly significant match [e values ranging from 1e-113 to 1e-112] to small GTPases from a variety of *Aspergillus *species. Overall, the deduced *P. marneffei *RanA peptide was 90% identical to these *Aspergillus *proteins over 214 amino acid residues. In addition, the *P. marneffei *RanA peptide was highly homologous to small GTPases from the dimorphic fungi *Candida albicans *and *C. immitis *(Fig. 5). Further analysis of the peptide sequence revealed a signature motif typical of Ran proteins from a variety of organisms.

## Discussion

A limited number of studies have focused on the molecular basis of phase transition in *P. marneffei*. Fewer, still, have employed broad screening approaches to study patterns of differential gene expression. Such investigations have utilized genomic [8,34] as well as proteomic strategies [31]. The latter study applied peptide mass fingerprinting in conjunction with two-dimensional difference gel electrophoresis to analyze selected proteins from fully differentiated mould and yeast cells. A number of proteins were putatively identified, including two encoded by previously characterized *P. marneffei *genes. This prior study also confirmed the up-regulated expression of these genes in the yeast phase. By comparison, the present investigation is the first to use MS sequencing in a proteomic approach to identify proteins expressed by *P. marneffei *during the early stages of phase transition. Our intent was to identify proteins that are more apt to play crucial roles in the induction of dimorphism rather than those associated with the latter stages of development.

In the recently published proteomic survey by Xi *et al*. [31] that compared mould and yeast phase proteins *of P. marneffei*, several thousand protein spots were reportedly resolved by two-dimensional difference gels. In contrast, we observed a few hundred proteins using 2DGE. The disparity between these data can be attributed to several factors. First, the prior study used a different strain of *P. marneffei *(IFM52703) than employed in the present investigation (strain F4; CBS 119456). Given the different geographic origins of each isolate (China and Thailand, respectively), there are likely to be innate differences in the genetic structure of these two strains that may be reflected in their protein profiles. Molecular typing studies of various *P. marneffei *isolates supports this premise [35-38]. In addition, the prior study used a broader pI range to resolve proteins that were subsequently detected with a reportedly more sensitive dye (CyDye) than we employed. Also, the earlier investigation extracted proteins from older cells than those we studied. These older cells are likely to contain a variety of different proteins than those found in the younger cultures employed in our investigation. In fact, the spectrum of selected proteins identified by Xi *et al*. [31] generally differed from those found in the present study. This may reflect a bias by both investigations in choosing prominent proteins for identification or technical limitations associated with the methodologies employed. Nonetheless, both studies did demonstrate the up-regulation of heat shock-related proteins in development of the yeast phase of *P. marneffei*, although the same proteins were not identified. Curiously, Xi *et al*. [31] observed that a heat shock-related protein homologous to the *A. fumigatus *mitochondrial Hsp70 chaperone (NCBI Accession No. XP_755328) was down regulated in the yeast phase of *P. marneffei *strain IFM52703. These same investigators noted that a cyclophilin-like protein (NCBI Accession No. XP_360696) was up-regulated in the yeast phase of the same strain. In contrast, our investigation identified a cyclophilin-like precursor that was expressed at roughly equivalent levels in the both the mould and yeast phases of *P. marneffei *strain F4.

One unique aspect of our investigation is that it represents a significant step in linking both genomic and proteomic strategies in an effort to decipher the molecular mechanisms of phase transition in a pathogenic fungus. A number of studies by Andrianopoulos and colleagues have focused on intracellular signaling pathways as they relate to morphogenesis in *P. marneffei *[12,22,23,28,29,39]. To date, using genetic approaches, they have identified several G-proteins and other DNA motifs that regulate conidiogenesis, cell polarity, germination, yeast growth, transcription, and other cellular developmental functions. However, none has yet been determined to be the key element in the induction of dimorphism although a published study by this group suggests that there does exist a yet unknown thermal sensing element [27]. Recently, a hybrid histidine kinase was determined to be a primary regulator of dimorphism and pathogenicity in *Blastomyces dermatitidis *and *Histoplasma capsulatum *via its ability to sense and respond to environmental stresses, including temperature [17,40]. Such observations may prompt genomic- and proteomic-based studies directed at discovering a similar mechanism in *P. marneffei*.

In the present study, we exploited the results of our proteomic study to clone the *P. marneffei RanA *gene. This particular gene was selected not only due to its conserved nature among eukaryotic organisms, but also because it encodes a small GTPase that apparently is up-regulated during the initial stages of yeast development. *RanA *orthologs in fungi facilitate the transport of materials across the nuclear membrane, help regulate mitotic activities, and have possible roles in the spatial and temporal organization of eukaryotic cells [41,42]. It is curious to note that the increased levels of the *P. marneffei *RanA protein correlate with the apparent co-ordination of cell division with nuclear division when this fungus is grown at 37°C (see [22]). These co-ordinated events generate uninucleate yeast cells. While direct evidence regarding the exact relationship of the *P. marneffei RanA *gene to phase transition remains to be discovered, it is clear that additional intracellular signaling mechanisms are involved in this process than currently described [12,22,23,28,29]. Moreover, the cloning of RanA has effectively demonstrated the linkage of both genomic and proteomic tactics in an effort to better understand the biology of *P. marneffei*. We have further exploited these tactics to clone additional cognate genes of proteins identified in this study. Using MS-derived peptide sequences to search a non-annotated version of the *P. marneffei *genome, we have now cloned genes encoding homologs of cyclophilin (*cypA*), RACK-1 (*cpcB*), ATP phosphoribosyltransferase (*hisA*), antioxidant protein LsfA (*lsfA*), and adenylate kinase (*adkA*) (NCBI Accession Nos. EU532183 to EU532187).

Finally, as might be expected, we identified several heat-shock/stress-related proteins that were expressed during yeast development in *P. marneffei*, including heat shock protein 30 (spot A3; Table 3) and the recently characterized heat shock protein 70 (spot D16; Table 3; [26]). Similar observations have been previously noted in both proteomic and genetic studies [31,34]. However, we have identified two other proteins of particular interest – Cdc48p and UDP-N-acetylglucosamine pyrophosphorylase. In eukaryotic cells, the former has a number of important functions in the proteasome including the eradication of misfolded proteins [43,44]. However, in the yeasts *Saccharomyces cerevisiae *and *Schizosaccharomyces pombe*, Cdc48p also appears to have essential roles in the initial commitment step of the cell cycle ('start') and in sister chromatid separation during mitosis [45,46]. With regard to UDP-N-acetylglucosamine pyrophosphorylase, this enzyme is essential to the synthesis of N-acetylglucosamine, the monomeric component of cell-wall chitin. Chitin is crucial to the integrity of the cell walls of fungi. Moreover, enzymes involved in chitin synthesis not only play significant roles in fungal dimorphism, but also have been determined to be virulence factors in a number of mycotic disease-causing agents [47-53]. The specific roles that Cdc48p and UDP-N-acetylglucosamine pyrophosphorylase fulfill in the phase transition of *P. marneffei *will require additional investigation. Conceivably, Cdc48p may have a function in linking cellular growth patterns with nuclear division during yeast development in *P. marneffei*, a process that has already been noted [22,23]. Likewise, the function of UDP-N-acetylglucosamine pyrophosphorylase clearly indicates that it has a role in cell-wall biosynthesis. Although the cell wall of the mould or yeast phases of *P. marneffei *has not been well characterized, there are obvious ultrastructural wall changes that occur during phase transition which undoubtedly involve specific patterns of chitin incorporation [18-21,54].

## Conclusion

We have employed proteomic methodologies to study the early molecular events associated with phase transition in the pathogenic fungus, *P. marneffei*. The information generated here provides the foundation for future work, both at the proteomic and genomic level, directed at characterizing the underlying mechanisms of cellular development in this fungus. We demonstrated the linkage between genomics and proteomics in this fungus by cloning and characterizing the *P. marneffei RanA *gene in which the increased levels of the corresponding protein correlate with the switch in development from a multinucleate mould to a uninucleate yeast. Potentially, the data generated by this and future investigations may identify potential targets for novel chemotherapeutic interventions that might be applied to treat diseases caused by this and other fungi. This proteomic strategy will be furthered when the fully annotated genome of *P. marneffei *is completed.

## Materials and methods

### Chemicals, reagents, and media

Unless otherwise noted, all chemicals, reagents, and media were obtained from Amresco, Inc. (Solon, OH), Bio-Rad, Inc. (Hercules, CA), Fisher Scientific (Pittsburgh, PA), or Sigma Chemical Co. (St. Louis, MO). The culture media, potato dextrose agar (PDA; Difco brand) and Sabouraud dextrose broth (SDB; Difco brand), were manufactured by Becton, Dickinson, and Co. (Sparks, MD). All solutions and media were prepared in distilled-deionized water (ddH_2_O).

### Fungal culture and inocula

The strain of *Penicillium marneffei *used in these investigations, F4 (CBS 119456), is a clinical isolate from a Thai AIDS patient at Maharaj Nakorn Chiang Mai Hospital. This strain, generously provided by Dr. Nongnuch Vanitanakom (Chiang Mai University, Thailand), was routinely maintained at 25°C on PDA. For long-term storage, conidial suspensions were stored either at room temperature in sterile ddH_2_O or at -80°C in sterile 15% (v/v) glycerol.

Experimental inocula consisted of conidial suspensions derived from strain F4 grown on PDA at 25°C for 8–10 days in 150 cm^2 ^cell culture flasks with vented caps. Conidia and accompanying hyphae were collected by gently scraping the culture surface after flooding it with 10 ml of sterile ddH_2_O. The resulting suspension was passed through a sterile glass wool separation unit to remove hyphal fragments. The separation unit consisted of a 1-inch wedge of glass wool (Corning, Acton, MA) placed between two screened caps (Bio-Rad) secured to each other by means of their screwed threads. The unit was wrapped in aluminum foil and sterilized by autoclaving before use. Subsequently, the unit was secured to the end of a sterile 50-ml conical, screw-capped centrifuge tube. The *P. marneffei *cell suspension was transferred into the top of the separation unit, then enclosed with the cap from the centrifuge tube. Next, conidia were isolated by centrifugation of the completed separation unit for 30 sec in a swinging bucket rotor at a relative centrifugal force of 150–200 × g. The fluid that passed through the unit contained >99.9% conidia, whereas the hyphae in the original suspension were retained by the glass wool (data not shown). The concentration of the conidial suspension, consisting mainly of individual cells, was determined using a hemocytometer.

### Experimental cultures and microscopy

Experimental cultures consisted of 50 ml SDB in 500-ml Erlenmeyer flasks inoculated to a final concentration of 1 × 10^7 ^conidia per ml. For each 50 ml SDB culture, a volume of the water suspension containing 5 × 10^8 ^conidia was collected by centrifugation (2,500 × g at 4°C for 15 min) in 15-ml conical, screw-capped tube. After decanting the supernatant, the conidial pellet was resuspended in a small portion (~3 ml) of the SDB (pre-warmed to either 25°C or 37°C, as appropriate) to be inoculated, then immediately transferred to the remaining media in the flask.

Individual experimental cultures, prepared as described above, were incubated in either a 25°C or a 37°C orbital water bath operating at 120 rpm. After 24 hours of incubation, the entire volume of culture fluid was centrifuged in pre-chilled (on ice) bottles at 17,600 × g for 15 min at 4°C. The resulting cell pellet was then washed twice by suspending the cell pellet in 30–50 ml ice-cold TE buffer (10 mM Tris, 1 mM EDTA, pH 8.0) prior to collecting the cells by centrifugation (17,600 × g for 15 min at 4°C). The washed cell pellet was kept on ice or frozen in small aliquots at -80°C prior to protein extraction.

For microscopy, aliquots of *P. marneffei *SDB cultures incubated at 25°C and 37°C were removed at various time points and the cells collected by centrifugation at 4°C for 5 min at 3000 × g. After removal of the supernatant, the cells were suspended in fixative (4% [w/v] *p*-formaldehyde in phosphate buffered saline, pH 7.4) for at least one hour. The fixed cells were mounted on slides and viewed using the differential-interference-contrast optics of an Olympus IX51 microscope (Olympus, Melville, NY). Digital photomicrographs were taken with an RKTE Spot Digital Camera and the associated Spot Software (Diagnostic Instruments, Sterling Heights, MI). Photomicrographs were also taken of *P. marneffei *slide cultures. These cultures, grown on PDA for 7–10 days at 25°C or 37°C, were prepared as previously described [55].

### Protein extraction

Cells frozen following collection from experimental cultures were thawed on ice prior to being processed. Thawed cells (approximately 400 mg wet weight), or those freshly collected from culture, were then transferred to an ice-cold 2 ml O-ring screw-capped microfuge tube prior to centrifugation (6000 × g for 10 min at 4°C). To this tube, an equal amount of acid-washed glass beads (0.5 mm dia; Biospec, Bartlesville, OK) was added, as well as 800 μl lysis buffer (20 mM Tris-HCl, pH 7.6, 10 mM NaCl, 0.5 mM sodium deoxycholate, 40 μl/ml of protease inhibitor cocktail). The cells were then broken using a Mini-BeadBeater^® ^(Biospec) operating at 5,000 rpm for a total of 4 min divided into 30 s increments. Sample tubes were maintained in an ice bath for at least 30 sec between increments. The resulting slurry was centrifuged at 6000 × g for 10 min at 4°C. The supernatant was transferred to a pre-weighed 1.5 ml microfuge tube and proteins were precipitated adding ice-cold trichloroacetic acid to a final concentration of 20% (v/v). After incubating the tube on ice for 20 min, a crude protein pellet was collected by centrifugation at 6000 × g for 20 min at 4°C. The supernatant was discarded and the pellet was washed three times with 500 μl ice-cold acetone. After each resuspension in acetone, the protein pellet was collected by centrifugation at 850 × g for 1 min at 4°C. Following the last acetone wash, the pellet was dried either by vacuum centrifugation or standing evaporation prior to being suspended in Modified Sample Buffer (MSB; 2 M thiourea, 7 M urea, 4% 3-[(3-Cholamidopropyl)dimethylammonio]-1-propanesulfonate (CHAPS), 1% dithiothreitol (DTT), 2% [v/v] carrier ampholytes, pH 3–10) according to final pellet weight (i.e., for a 5–15 mg pellet, 500 μl MSB was used; for a 16–30 mg pellet, 750 μl MSB was used). All samples were stored at -80°C.

### Protein quantification

A modified Bradford Assay was used to determine the concentration of protein in each sample [56]. Each reaction tube contained 80 μl water, 10–20 μl 0.1 M HCl, 10 μl 2DE buffer (8.4 M urea, 2.4 M thiourea, 5% CHAPS, 25 mM spermine base, 50 mM DTT; [57]), and 4.0 ml of Bradford dye (0.01% Coomassie Brilliant Blue G-250, 8.5% [v/v] phosphoric acid). A standard curve was established using serial dilutions of bovine serum albumin. Sample concentrations were determined using 5–10 μl of protein extract added to a reaction tube. The absorbencies of all reaction tubes were recorded at 595 nm.

### Two-dimensional gel electrophoresis

Electrophoresis in the first dimension employed 17 cm immobilized pH gradient (IPG) strips of various pI ranges (Bio-Rad) that were passively rehydrated with 300 μl rehydration buffer (8 M urea, 1% CHAPS, 15 mM DTT, 0.2% [v/v] BioLytes, 0.001% bromophenol blue). Subsequently, IPG strips were loaded with 250 μg protein prior to isoelectric focusing using a Protean IEF Cell (Bio-Rad) at 20°C for 60,000 V-hr. The IPG strips were then sequentially washed with equilibration buffers I (6 M urea, 2% sodium dodecyl sulfate (SDS), 0.375 M Tris-HCl pH 8.8, 20% [v/v] glycerol, 130 mM DTT) and II (6 M urea, 2% SDS, 0.375 M Tris-HCl pH 8.8, 20% [v/v] glycerol, 135 mM iodoacetamide) for 10 min each using gentle agitation.

For second dimension electrophoresis, the equilibrated IPG strips were rinsed with Tris-Glycine-SDS (TGS) buffer (25 mM Tris base, 192 mM glycine, 0.1% SDS) prior to being loaded onto 8% to 12% polyacrylamide gels (20 cm × 20 cm) prepared in electrophoresis buffer (0.375 M Tris [pH 8.8], 0.1% SDS). The strips were sealed in place using an agarose overlay (0.5% low melt agarose in TGS buffer with 0.001% bromophenol blue). The gels were loaded into a Protean II xi cell or a Protean Plus Dodeca Cell (both Bio-Rad). A constant current of 10–24 mA per gel was applied to the Protean XL system while a constant 200 V was applied to the Dodeca Cell apparatus.

### Image analysis

Following electrophoresis in the second dimension, the gels were placed in a fixing solution (40% methanol, 10% acetic acid; v/v) for one hour. This solution was replaced with SYPRO Ruby protein gel stain (Bio-Rad) for 4 – 24 hrs under gentle shaking conditions. Finally, the gels were rinsed in ddH_2_O with shaking for 15 min prior to visualization under UV illumination at 365 nm using a Molecular Imager ChemiDoc XRS system (Bio-Rad).

Digital images of 2DGE gels from given resolution parameters (i.e., acrylamide concentration and pI range) were generated for each of three or more independent cultures per incubation temperature (25°C or 37°C). The digital images of these three gels were used in conjunction with PDQuest 2-D Analysis Software (Bio-Rad) to create one match set per experimental condition. The master of each match set resulting from different experimental conditions was then subjected to further analysis using PDQuest. Differences in levels of expression, founded upon spot staining intensity, were assessed using three-dimensional contour maps. For data presentation, all digital images and contour maps were processed using PowerPoint (Microsoft Corp., Seattle, WA) and PhotoStudio (Arcsoft, Fremont, CA) software.

### Protein isolation and preparation for mass spectrometry

Based upon image analysis results, proteins of interest were isolated for sequencing by MS as described below. Specifically, cell extracts were subjected to 2DGE as described above except IPG strips were loaded with ≥ 400 μg of protein prior to first-dimension separation. After resolving proteins in the second dimension, gels were placed in 500 ml fixing solution (50% ethanol, 10% acetic acid; v/v) for one hour at room temperature. The fixing solution was then replaced with 500 ml of washing solution (50% methanol, 10% acetic acid; v/v) and the gel was incubated overnight at room temperature. The fixed gel was subsequently stained in fresh SYPRO Ruby stain for at least three hours at room temperature, then incubated in water for 15 min with gentle agitation. Finally, the gel was transferred to storage solution (5% [v/v] acetic acid) for one hour or more before visualizing the protein profiles using the ChemiDoc system.

Protein spots of interest were excised directly by hand from illuminated SYPRO Ruby-stained gels using a sterile Pasteur pipette or a 2500 μl pipette tip. The gel piece was then expelled into a sterile microfuge tube and covered with 5% (v/v) acetic acid. The samples were then submitted to the Ohio State University Mass Spectrometry and Proteomics Facility (Columbus, OH; ) for sequencing by MS.

The acrylamide-embedded protein samples submitted for MS sequencing were processed by the following procedure. First, samples were digested with sequencing grade trypsin (Promega, Madison, WI) or sequencing grade chymotrypsin (Roche, Indianapolis, IN) using the Montage In-Gel Digestion Kit (Millipore, Bedford, MA) following the recommended protocols. Briefly, samples were trimmed as close as possible to minimize background polyacrylamide material, then washed for one hour in a methanol/acetic acid solution (50% methanol: 5% acetic acid; v/v). The wash step was repeated once before gel pieces were dehydrated in acetonitrile. Subsequently, the protein/acrylamide samples were rehydrated in a DTT solution (5 mg/ml in 100 mM ammonium bicarbonate) for 30 minutes prior to the addition of iodoacetamide (15 mg/ml in 100 mM ammonium bicarbonate). The samples were incubated in the dark for 30 min before sequential 5 min washes in acetonitrile and 100 mM ammonium bicarbonate. Again, the samples were vacuum dried, then rehydrated in 50 μl of 50 mM ammonium bicarbonate containing either sequencing grade modified trypsin or chymotrypsin at 20 μg/ml. After 10 min of incubation, an additional 20 μL of 50 mM ammonium bicarbonate was added to the samples that were then incubated overnight at room temperature. Finally, the peptides were extracted several times from the polyacrylamide using an acetonitrile/formic acid solution (50% acetonitrile: 5% formic acid; v/v). The extracts were pooled, then concentrated under vacuum to a final volume of approximately 25 μl.

### Mass spectrometry

Capillary-liquid chromatography-nanospray tandem MS (Nano-LC/MS/MS) was performed on a Thermo Finnigan LTQ mass spectrometer equipped with a nanospray source operated in positive ion mode. The LC system was a UltiMate™ Plus system (Dionex, Sunnyvale, CA) with a Famos autosampler and Switchos column switcher. Solvent A was water containing 50 mM acetic acid and solvent B was acetonitrile. Five microliters of each sample was first injected on to the trapping column, then washed with 50 mM acetic acid. The injector port was switched to inject and the peptides were eluted off of the trap onto the column. A 5 cm 75 μm ID ProteoPep II C18 column (New Objective, Woburn, MA) packed directly in the nanospray tip was used for chromatographic separations. Peptides were eluted directly off the column into the LTQ system using a gradient of 2–80% B over 50 minutes, with a flow rate of 300 nl/min. A total run time was 60 minutes. The scan sequence of the mass spectrometer was programmed for a full scan, a zoom scan to determine the charge of the peptide, and a MS/MS scan of the most abundant peak in the spectrum. Dynamic exclusion was used to exclude multiple MS/MS of the same peptide.

Data processing was performed following recommended guidelines [58]. Sequence information from the MS/MS data was processed by converting the raw data files into a merged file (.mgf) using MGF creator (merge.pl, a Perl script) with first scan number, last scan number, number of intermediate scans, minimum number of grouped scans and minimum number of ions set to blank, blank, 1, 0, and 8, respectively. The resulting .mgf files were searched using Mascot Daemon (version 2.2.1; Matrix Science, Boston, MA) against the National Center for Biotechnology Information (NCBI; ) databases (NCBI nr). These databases contained in excess of 6 × 10^6 ^sequences comprised of more than 2 × 10^9 ^residues. Selected files were subjected to a more narrow search using the NCBI nr database limited to the taxon Fungi (>3 × 10^5 ^sequences). These databases were employed because a completely sequenced and annotated genome of *P. marneffei *has yet to be publicly released. The mass accuracy of the precursor ions were set to 2.0 Da given that the data was acquired on an ion trap mass analyzer and the fragment mass accuracy was set to 0.5 Da. Considered modifications (variable) were methionine oxidation and carbamidomethyl cysteine. Two missed cleavages for the enzyme were permitted. Peptides with a score less than 20 were filtered and proteins were identified having a significance threshold of p < 0.05. Protein identifications were checked manually and proteins with a Mascot score of 50 or higher with a minimum of two unique peptides from one protein having a *-b *or *-y *ion sequence tag of five residues or better were accepted.

### Amplification and characterization of the *P. marneffei RanA *gene

Oligonucleotide primers (RAN-1F: 5'-ACYTTCAAGCTCGTSCTCGT-3'; RAN-1R: 5'-TAGTTGGACYTGGCSGAGAT-3'; Integrated DNA Technologies, Coralville, IA) were designed to the putative *P. marneffei *RanA peptide sequences identified by MS (spot A1 [spi1, *Schizosaccharomyces pombe*]; see Table 3). These primers were then used in a polymerase chain reaction (PCR) to amplify a fragment of the cognate *RanA *gene. The PCR mixture consisted of AmpliTaq Gold PCR Master Mix (Applied Biosystems, Foster City, CA) containing 0.2 μM of each primer and approximately 300 ng of *P. marneffei *genomic DNA isolated as previously described [59]. The amplification reaction was conducted with a DNA Engine Peltier Thermal Cycler (Model PTC-200; Bio-Rad) programmed as follows: 95°C for 9.5 min; 35 cycles 95°C for 30 sec, 54°C for 30 sec, 72°C for 1 min; 72°C for 7 min; held at 4°C indefinitely. Amplification of the appropriately-sized PCR product was confirmed by gel electrophoresis [60] prior to its cloning into plasmid pCR4-TOPO (TOPO TA Cloning Kit for Sequencing; Invitrogen, Carlsbad, CA). The nucleotide sequence of the cloned insert was then determined using M13 forward and M13 reverse primers (Invitrogen) in conjunction with the Dye Terminator Cycle Sequencing Quick Start Kit and the CEQ 2000 DNA sequencer (both Beckman Coulter, Fullerton, CA). The sequence data files were initially analyzed using 4 Peaks software , which includes BLAST search capabilities (; [61,62]). Subsequently, the 5' end of putative *RanA *gene was extended using the GeneRacer RACE Ready cDNA kit (Invitrogen). The RNA for this latter procedure was isolated from 37°C-grown cultures of *P. marneffei *employing the RNeasy Mini kit (QIAGEN, Valencia, CA). The 3' end of the *P. marneffei RanA *gene was cloned and sequenced using the DNA Walking SpeedUp™ Premix Kit (version 2.1; Seegene, Inc., Seoul, Korea) in accord with the manufacturer's recommendations. Finally, a 1525 bp DNA fragment containing the entire *RanA *gene was amplified from genomic DNA using specific primers (PmRan-F: 5'-CACAAGCTTGCGACTCTCAG-3'; PmRan-R: 5'-TCTGGACCATGTGGAGAAGA-3') in a PCR employing the same conditions described above. The resulting PCR product was cloned and its nucleotide sequence confirmed as described above using additional primers designed to the previously generated data. The confirmed nucleotide sequence of the *P. marneffei RanA *gene was deposited in GenBank (; NCBI Accession No. EF519305). The deduced amino acid sequence of the *P. marneffei *RanA protein, derived from this data using a world-wide-web-based translation program  was subjected to a BLAST search of protein databases  as well as a PROSITE motif pattern search . Peptide sequence alignments were performed using Clustal W ; [63]).

## Competing interests

The authors declare that they have no competing interests.

## Authors' contributions

JMC helped design experiments, generated protein profiles, acquired and interpreted data, performed the gene cloning and sequencing experiments, and assisted in the drafting and review of the manuscript. ERT and HRT conducted protein profiling experiments as well as acquired and interpreted data acquisition. Significant contributions in generating protein profiles, gene cloning and sequencing, and data analysis were performed by JLB, JLF, ZMO, MMP, WTR, OVV, and TJF. TDK, GRW, and CRC conceived of the study, participated in its design and coordination, interpreted the data, and drafted the manuscript. All authors read and approved the final manuscript.
